# The role of the CCN family of proteins in blood cancers

**DOI:** 10.1007/s12079-016-0342-x

**Published:** 2016-08-03

**Authors:** Lisa Judith Crawford, Alexandra Elizabeth Irvine

**Affiliations:** Haematology Research Unit, Centre for Cancer Research and Cell Biology, Queen’s University Belfast, Room OG/013, Belfast, BT9 7BL Northern Ireland

**Keywords:** Blood cancer, Bone marrow microenvironment, Haematopoiesis, Leukaemia, Lymphoma, Stem cells

## Abstract

Haematopoiesis is the term used to describe the production of blood cells. This is a tightly regulated hierarchical system in which mature circulating blood cells develop from a small population of haematopoietic stem (HSC) and progenitor cells within the microenvironment of the bone marrow. Molecular and genetic abnormalities arising in these stem cells lead to a block in the normal programme of proliferation and differentiation and result in the development of the blood cancers known as the leukaemias and lymphomas. Recently the regulatory role of the bone marrow microenvironment or niche has also become increasingly recognised. The interface between the bone and bone marrow (endosteum) and the region surrounding the blood vessels (perivascular) provide distinct niches harbouring quiescent HSC or proliferative HSC respectively. Current chemotherapeutic regimes can often successfully target the proliferative HSC but disease relapse occurs due to residual quiescent HSC. Understanding these developmental and regulatory processes and the associated cell communication mechanisms are thus crucial to the development of new treatment strategies. The CCN family of proteins have been recognised to play a key role in all aspects of haematopoiesis.

## Normal blood cell development

### Bone marrow microenvironment

**Fig. 1:**
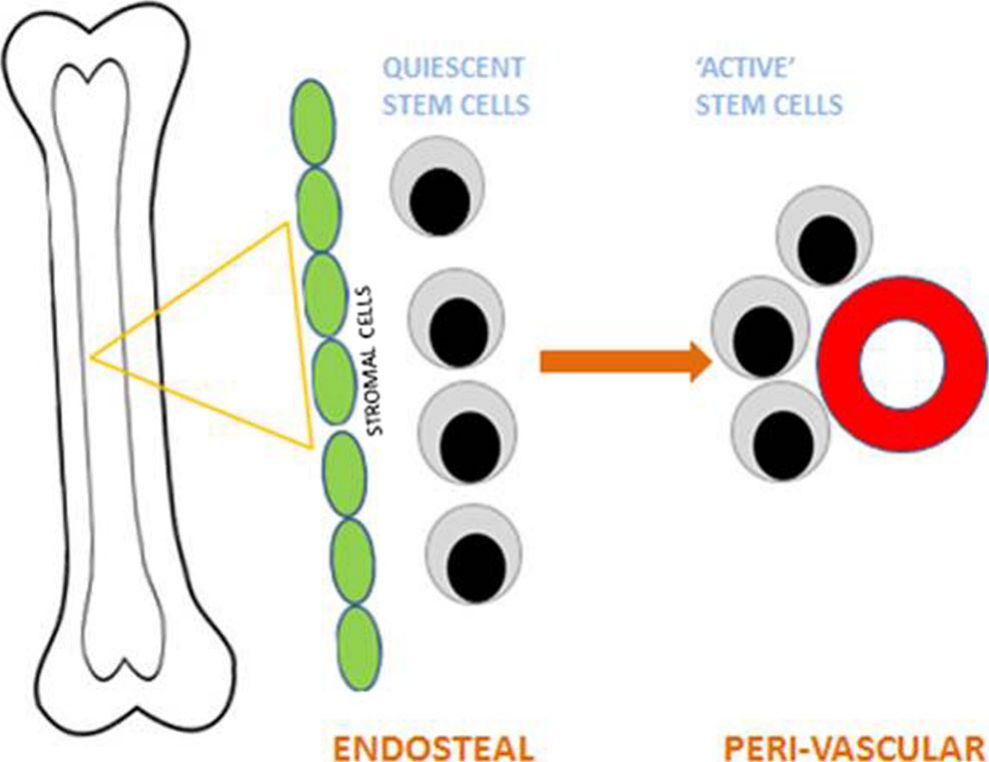
The Bone Marrow Microenvironment. The marrow microenvironment consists of two distinct niches: the endosteal compartment which harbours quiescent stem cells and the region surrounding the blood vessels, peri-vascular, which harbours proliferative stem cells

Normal adult blood cell production (haematopoiesis) takes place mainly within the bone marrow. It is now acknowledged that this microenvironment plays a key role in maintaining the balance between proliferation and differentiation (Morrison and Scadden 2014; Anthony and Link 2014). Specific regions or niches within the marrow are associated with distinct stem cell populations and this in turn is due to the stromal cell populations present and the regulatory molecules which they produce. The earliest stem cells, known as quiescent cells, are generally found at the interface between the bone and bone marrow or endosteal region. In contrast, the more proliferative stem cells are usually found in the perivascular region (Ho et al. 2015a). Matricellular proteins, including CCN family members, play a key regulatory role in the bone marrow microenvironment (McCallum and Irvine 2009; Cheung et al. 2014; Johnson et al. 2014a) (Fig. 1).

### Stem and progenitor cells

**Fig. 2:**
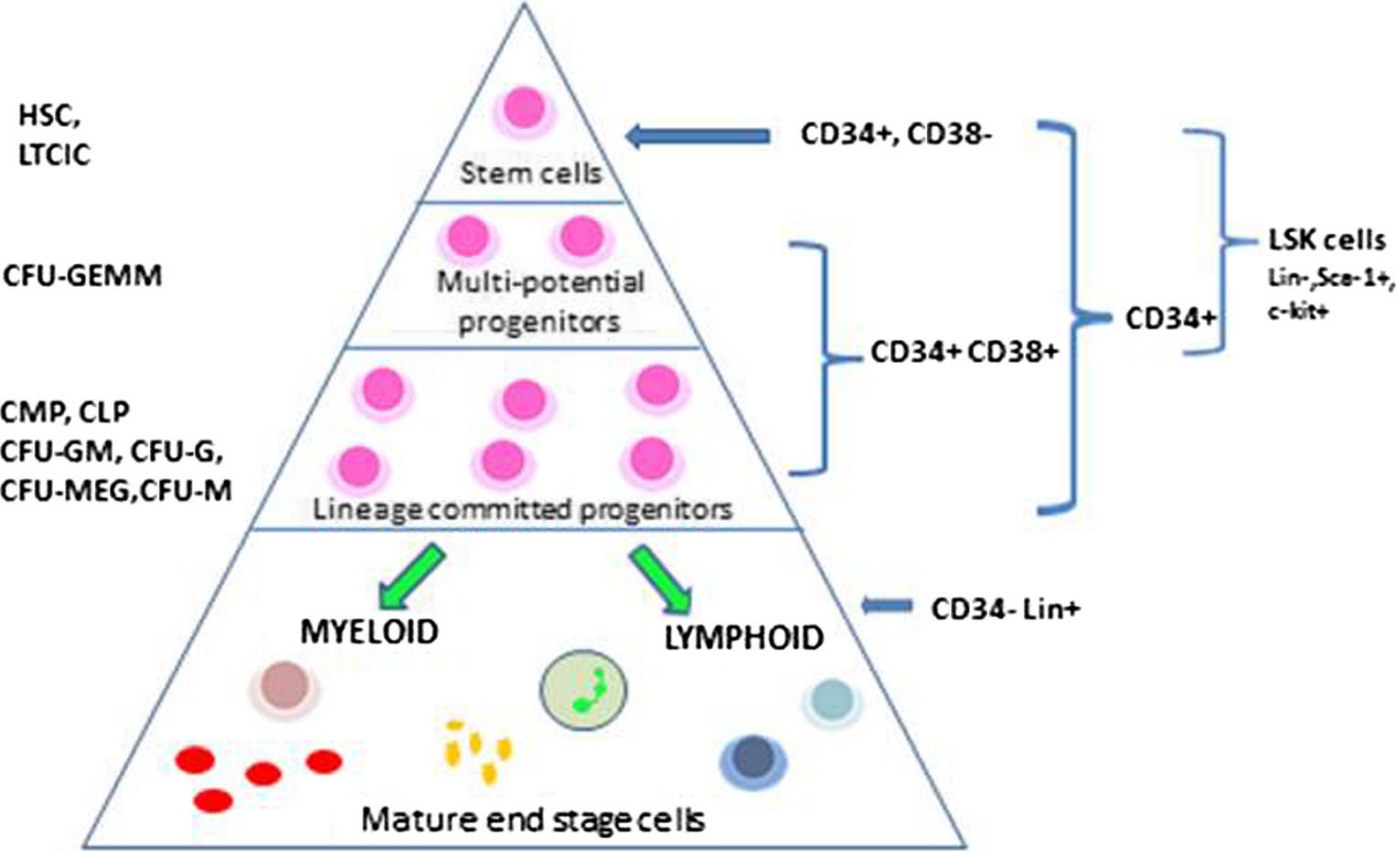
Haematopoiesis. Haematopoiesis is a hierarchical structure in which a small number of stem cells go through a programme of proliferation and differentiation to produce the mature cells which circulate in the blood stream. The different stem cell populations may be named on the basis of the functional clonogenic assays in which they were first described (left hand side of diagram) or by their surface antigen expression (right hand side of diagram)

Haematologists have been interested in characterising the developmental stages of blood cell production for many years. Early work focussed on the use of clonogenic assays and the terminology derived from this still persists today (Ho et al. 2015). The prefix CFU denotes colony forming unit and the suffix (G: granulocyte, M: macrophage, E: erythroid, MEG: megakaryocyte) distinguishes the lineage of the cells making up the clone. Developments in immunological classification and flow cytometry allowed us to categorise these progenitor cells further and provided an opportunity to sort and work with purer populations of cells. Knowledge of surface antigen expression has facilitated further discrimination between stem and progenitor cell populations (Ho et al. 2015). Our current concept of haematopoiesis is that of a hierarchical structure in which a small number of multipotent stem cells develop along specific pathways and differentiate into the mature circulating blood cells (Fig. 2).

### The role of the CCN family of proteins in haematopoiesis

There are limited published studies on the role of CCN family members in haematopoiesis and fewer still which have examined the specific role of the microenvironment. Studies with mouse bone marrow (Cheung et al. 2014) have shown that CCN2 is expressed by bone marrow stromal cells although it was barely detectable in unfractionated cells per se. A chimaeric fetal liver transplant model and culture systems were used to show that CCN2 promotes B cell lymphopoiesis in the presence of IL-7. CCN3 expression has also been reported in mouse bone marrow both in the endosteal region near the epiphysis and between the trabecular bone and microvasculature. It was suggested that these CCN3 expressing cells represented both haematopoietic and stromal cell populations (Katsube et al. 2009).

**Fig. 3:**
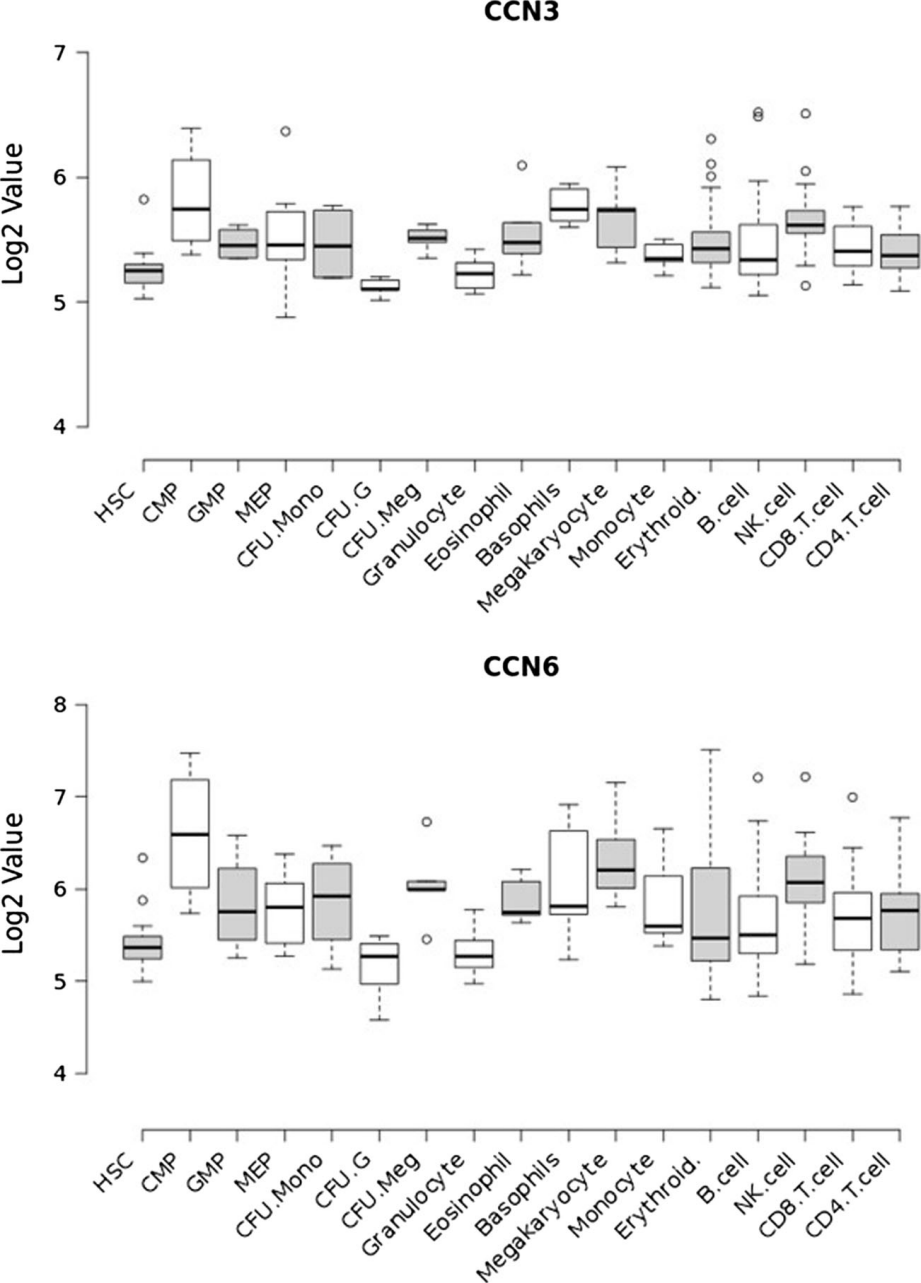
CCN expression in normal haematopoiesis. CCN3 and CCN6 gene expression in enriched populations of haematopoeitic cells (Data from GSE24759). HSC – haematopoietic stem cell; CMP – common myeloid progenitor; GMP – granulocyte-monocyte progenitor; MEP – megakaryocyte-erythroid progenitor; CFU-Mono – monocyte colony forming units; CFU-G – granulocyte colony forming units; CFU-Meg – megakaryocyte colony forming units

It is difficult to obtain normal bone marrow for research studies and if one fractionates the sub-populations of stem and progenitor cells there are very few cells to work with. There are thus few publications in this area. BloodSpot is a publically available database of mRNA from a number of curated data sets encompassing expression profiles in normal and malignant haematopoiesis (Bagger et al. 2013; Bagger et al. 2012; Bagger et al. 2016). The expression profile of CCN family members was analysed in the Differentiation Map (DMAP) data set which is comprised of purified populations of haematopoietic stem cells, multiple progenitor cells and mature differentiated blood cells (211 samples). Expression of CCN1, CCN2, CCN4 and CCN5 remained largely constant across the different cell populations. CCN3 and CCN6 followed similar patterns of expression and were observed to be expressed most highly in progenitor and mature cells of the myeloid lineage (with the exception of granulocytes), suggesting an association of these CCN members with myeloid differentiation. None of this data has yet been validated at the protein level (Fig. 3).

Most reports of CCN expression use enriched rather than pure populations of cells and data generated must be interpreted in the appropriate context. Similarly, the limited number of cells obtainable for study mean that most investigators focus on the role of a single CCN family member and mechanistic studies are only available in malignant pathologies where there are good cell line models available. CCN2 and CCN3 are the only family members which have been studied extensively in normal haematopoiesis.

CCN2 expression in normal cells has been studied in both the adult and paediatric setting. CCN2 was found to be negative by PCR in mononuclear cells from 120 children (age 1.8–15.8 years) with insulin-dependent diabetes mellitus (IDDM) and CD34+ cells from 100 cord blood samples (Vorwerk et al. 2000). Similarly, more recent investigations of adult mononuclear cells from normal bone marrow (10 samples), peripheral blood (10 samples) and CD34+ enriched cells from bone marrow (7 samples) or peripheral blood (9 samples) confirmed this lack of expression using microarrays and quantitative PCR (Sala-Torra et al. 2007).

CCN3 expression has been studied in normal human adult bone marrow and peripheral blood (McCallum et al. 2006) as well as cord blood (Gupta et al. 2007). In adults we detected CCN3 at all stages of development within the myeloid lineage; CD34+ progenitors, mononuclear cells (MNC) and neutrophils derived from normal bone marrow (NBM) and normal peripheral blood (nPB). We found the highest expression in CD34+ progenitors and MNC cells whilst expression was lower in mature neutrophils.

Human cord blood (CB) is also a recognised source of haematopoietic progenitor cells although these cells differ subtly from those found in bone marrow (Moretta et al. 2016; Notta et al. 2016). These disparities may be a reflection of the contrast in microenvironment in which CB stem cells develop (Stojko et al. 2016; Beksac 2016). Gupta et al. (Gupta et al. 2007) isolated primitive progenitors from human cord blood and found that, in contrast to the adult setting, CCN3 expression was restricted to the CD34+ progenitor cell population. They then used a CCN3 knockdown strategy to show that CCN3 was required to maintain long term culture initiating cell (LTC-IC) cultures, the most primitive haematopoietic progenitors; colony forming capacity of less primitive progenitors was enhanced by this strategy, but these progenitors failed to generate secondary colonies. The CCN3 knockdown cells had reduced Hes1 activity and a diminished response to Jagged1, consistent with reported links between CCN3 and the Notch signaling pathway (Suresh and Irvine 2015). These findings suggest that CCN3 is required for self-renewal and distinguishes alterations in response to CCN3 within subpopulations of primitive stem cells. It also highlights the importance of CCN3 and Notch relationship within haematopoietic development.

Studies in mice have shown that CCN3 regulates long term repopulating activity of HSCs through binding to integrin αvβ3 (Ishihara et al. 2014). Thrombopoietin (TPO) mediates this integrin binding and also stimulates the expression of CCN3 in HSCs. In contrast, stem cell factor (SCF) co-cultured with CCN3 had no effect on HSC function and inhibited expression of CCN3. This study establishes that CCN3 expression in HSCs is strongly influenced by cytokine stimulation and this in turn is a reflection of the microenvironment in which the cells live.

Further evidence for the role of cytokines in CCN3 regulation comes from the work of Kimura (Kimura et al. 2010). They used a microarray approach to look for target genes of STAT5A/B activated by cytokines in murine HSCs and progenitors. They showed that IL-3 induced binding of STAT5A/B to a γ-interferon activated sequence site in the CCN3 gene promoter region. This paper provides additional confirmation that CCN3 is a cytokine inducible gene and demonstrates that it is a direct target of STAT5A/B. The STAT signalling pathway plays a key role in haematopoiesis and it is widely reported as being implicated in a wide range of haematological neoplasms, particularly those of myeloid origin (Chougule et al. 2016; Balligand et al. 2016; Koschmieder et al. 2016; Miltiades et al. 2016; Muller et al. 2016; Wang and Bunting 2016; Xu et al. 2016).

## Blood cancers

### CCN1 and multiple myeloma

Multiple myeloma (MM) is characterised by the accumulation of malignant plasma cells in the bone marrow (BM) leading to the overproduction of monoclonal immunoglobulins, osteolytic bone lesions, renal disease and immunodeficiency. MM is usually preceded by the premalignant conditions monoclonal gammopathy of undetermined significance (MGUS) and/or smouldering multiple myeloma (SMM). Interactions between MM cells and the BM microenvironment are well-established to play a role in supporting the growth and survival of MM cells, as well as enhancing bone resorption through increased osteoclast activity and suppression of osteoblast activity (Kawano et al. 2015).

CCN1 is known to play a role in bone remodelling, primarily through stimulation of osteoblast differentiation (Kawaki et al. 2011) and inhibition of osteoclastogenesis (Crockett et al. 2007) and this has led a number of groups to investigate its role in MM. Two studies have recently reported that mesenchymal cells in the BM microenvironment of MM patients express CCN1 (Dotterweich et al. 2014; Johnson et al. 2014). Dotterwich and colleagues (Dotterweich et al. 2014) reported that co-culture of a MM cell line with primary mesenchymal cells from healthy donors promotes splicing, transcription and expression of CCN1 in MM cells. They further showed that exogenous CCN1 enhanced survival of a MM cell line and suggest that CCN1 may support MM cell viability and associated bone disease.

In contrast to this, Johnson (Johnson et al. 2014) reported that recombinant CCN1 decreased the growth of a MM cell line at high concentrations and overexpression of CCN1 in MM cells reduced tumour growth, inhibited osteoclastogenesis and stimulated osteoblastogenesis in a mouse model of MM. Furthermore, the authors found that CCN1 levels in the BM microenvironment could be a biomarker for MM progression. High levels of CCN1 in patients with MGUS or SMM were found to be associated with a longer time to progression to active MM, and elevated levels of CCN1 in MM patients in remission were associated with superior progression-free and overall survival. This study suggests that increasing CCN1 levels in the BM microenvironment may offer an approach to delay the progression of MGUS/SMM to active MM, as well as enhancing survival of patients with active myeloma.

In support of this theory, Li (Li et al. 2012) reported that intrabone injection of mesenchymal stem cells, expressing high levels of bone remodelling proteins such as CCN1, prevented MM-induced bone disease, promoted osteoblast maturation, suppressed osteoclast formation and inhibited MM cell growth in a preclinical model of MM. While the role of CCN1 in MM is not yet fully unravelled, overall the evidence to date suggests that CCN1 could provide a therapeutic strategy for treating MM bone disease.

### CCN1 and acute myeloid leukaemia

Acute Myeloid Leukaemia (AML) is a term used to represent a group of leukaemias in which there is a block in differentiation in the myeloid lineage of cells. These leukaemias are the result of malignant transformation in haematopoietic stem or progenitor cells and are characterised by the presence of 20 % or more immature blast cells in the blood or bone marrow at diagnosis. AML is classified into specific subtypes based on cytogenetic and molecular characteristics defined by the World Health Organisation. This classification provides prognostic information regarding poor, intermediate and favourable risk groups (Arber et al. 2016). It is therefore important in all studies of AML to know the relevant classification of the patients being studied.

There have been reports of a role for CCN1 in both bone marrow microenvironment and stem cells in AML. It has been suggested that bone marrow stromal cells may play a role in chemotherapy resistance. Studies with bone marrow stromal cell lines showed high levels of expression of CCN1 and knockdown of CCN1 using antibodies or RNAi led to increased sensitivity to chemotherapy in AML-stromal cell co-cultures (Long et al. 2015). Further investigations found that CCN1 signalling was mediated by Spleen Tyrosine Kinase (SYK) which acts downstream of CCN1 and contributes to chemo-resistance. This work was carried out using NB4 and THP1 cell lines which represent adult pro-myelocytic (favourable risk) and pediatric monocytic (intermediate risk) AML; details of the primary AML cells used were not given (Long et al. 2015). It is therefore difficult to ascertain how applicable these observations are to other AML subtypes.

The largest cohort of AML patients on which CCN1 expression data is available (500 patients) is from the MILE (**M**icroarray **I**nnovations in **LE**ukemia) study (Kohlmann et al. 2008). This database contains over 500 AML samples classified into 6 clinically recognised subtypes and identified increased expression of CCN1 in two subgroups, t(8;21) and complex karyotype, associated with favourable and unfavourable risk, respectively; array data only is available for this study. CCN1 protein expression has also been examined in a panel of leukaemia cell lines and found to be increased in two AML cell lines representing a pediatric myelocytic (favourable risk) and adult monocytic (unfavourable risk) AML. Knockdown of CCN1 using antibodies or siRNA suppressed proliferation and increased apoptosis suggesting that CCN1 was acting as a tumour promoter in AML (Niu et al. 2014). Primary AML bone marrow cells showed similarly increased levels of CCN1 although no patient details were given and there was no apparent association with risk group. Mechanistic studies demonstrated that CCN1 acts through the MEK/ERK pathway leading to upregulation of cMyc and Bcl-x_L_ and downregulation of BAX (Niu et al. 2014).

### CCN2 and acute lymphoblastic leukaemia

Acute Lymphoblastic Leukaemia (ALL) is the most common leukaemia found in children but it is also found in adults where it has a poor prognosis. Research in this field is focussed on finding new prognostic biomarkers and alternative therapeutic targets (Arber et al. 2016; He et al. 2016).

CCN2 has been found to be overexpressed in both adult and paediatric ALL compared to normal HSCs (Sala-Torra et al. 2007; Vorwerk et al. 2000). A role for CCN2 as a tumour marker was first reported by Vorwerk et al. who demonstrated by RT-PCR that it was specifically expressed in malignant lymphoblasts in childhood ALL (Vorwerk et al. 2000). Further work using quantitative PCR and microarrays confirmed high CCN2 expression in lymphoblasts in adult ALL and allowed discrimination from Acute Myeloid Leukaemia (AML)(Sala-Torra et al. 2007). Hypo-methylation of the CCN2 gene locus has been demonstrated to be a common feature in paediatric ALL and it has been suggested that it is a necessary but not sufficient pre-requisite for its deregulated transcription (Welch et al. 2013).

It has been shown that CCN2 expression is associated with poor prognosis (Sala-Torra et al. 2007; Wells et al. 2016). Quantitative PCR carried out on 79 adult ALL cases found CCN2 expression was associated with unfavourable cytogenetics and a worse overall survival (Sala-Torra et al. 2007). Pre-clinical studies with ALL cell lines and mouse models found that in vitro knock down of CCN2 or treatment with anti-CCN2 antibody could prolong survival (Lu et al. 2014). Recent work with mice has elucidated the potential underlying mechanism for this effect. Injection of NOD/SCID mice with high or low CCN2- expressing cells found that elevated levels led to reduced survival and that CCN2 had affected the bone marrow microenvironment causing increased extra-cellular matrix deposition (Wells et al. 2016). Together these studies suggest that CCN2 may provide a novel therapeutic target in ALL.

### CCN2 and lymphoma

The Lymphomas are a group of blood cancers in which malignant lymphocytes accumulate in the lymph nodes causing characteristic swelling referred to as lymphadenopathy. Occasionally the malignant cells spill into the blood and the disease enters what is referred to as a ‘leukaemic phase’. In these disorders the relevant microenvironment is essentially the lymph node (Burger et al. 2009). There are two major sub-divisions of lymphoma called Hodgkins and non-Hodgkins based on the presence or absence of Reed Sternberg cells, a type of neoplastic B cell. Hodgkins disease generally has a good prognosis whereas the non-Hodgkins lymphomas encompass a diverse group of disorders with poorer outcome.

Gene expression studies carried out on patients with large B-cell lymphoma, a non-Hodgkins lymphoma, identified an expression signature associated with good prognostic outcome (Lenz et al. 2008). The authors found that stromal cells expressing genes encoding components of the extracellular matrix, including CCN2, were found in patients with improved survival. Their findings support the importance of targeting the microenvironment for improved therapeutic efficacy (Burger et al. 2009).

CCN2 has been shown to be highly expressed in cells from patients with Hodgkins (44 patients) compared to non-Hodgkins (32 patients) lymphoma (Birgersdotter et al. 2010). Increased expression was demonstrated at both the RNA and protein level and it was suggested that this was associated with the high incidence of fibrosis in these patients. Elevated CCN2 expression has also been demonstrated in cells from patients with peripheral T cell lymphoma, not otherwise specified, compared to normal peripheral blood T cells. In this case data was reported from microarray analysis and not further validated (Mahadevan et al. 2005).

### CCN3 and chronic myeloid leukaemia

Chronic Myeloid Leukaemia (CML) is the only blood cancer which is characterised by a single specific genetic marker, the Philadelphia chromosome. This is caused by a reciprocal translocation between chromosomes 9 and 22 and leads to the formation of the bcr-abl fusion protein associated with elevated tyrosine kinase activity. Tyrosine kinase inhibitors have been developed as targeted therapy for CML and have been successful in obtaining long term remissions in patients. Nevertheless, many patients become resistant to these inhibitors and it is acknowledged that the leukaemia initiating cell remains. Efforts are now being made to find targets other than the kinase activity and to understand the relationship of the leukaemic stem cell with the surrounding microenvironment (Hughes and Ross 2016).

**Fig. 4:**
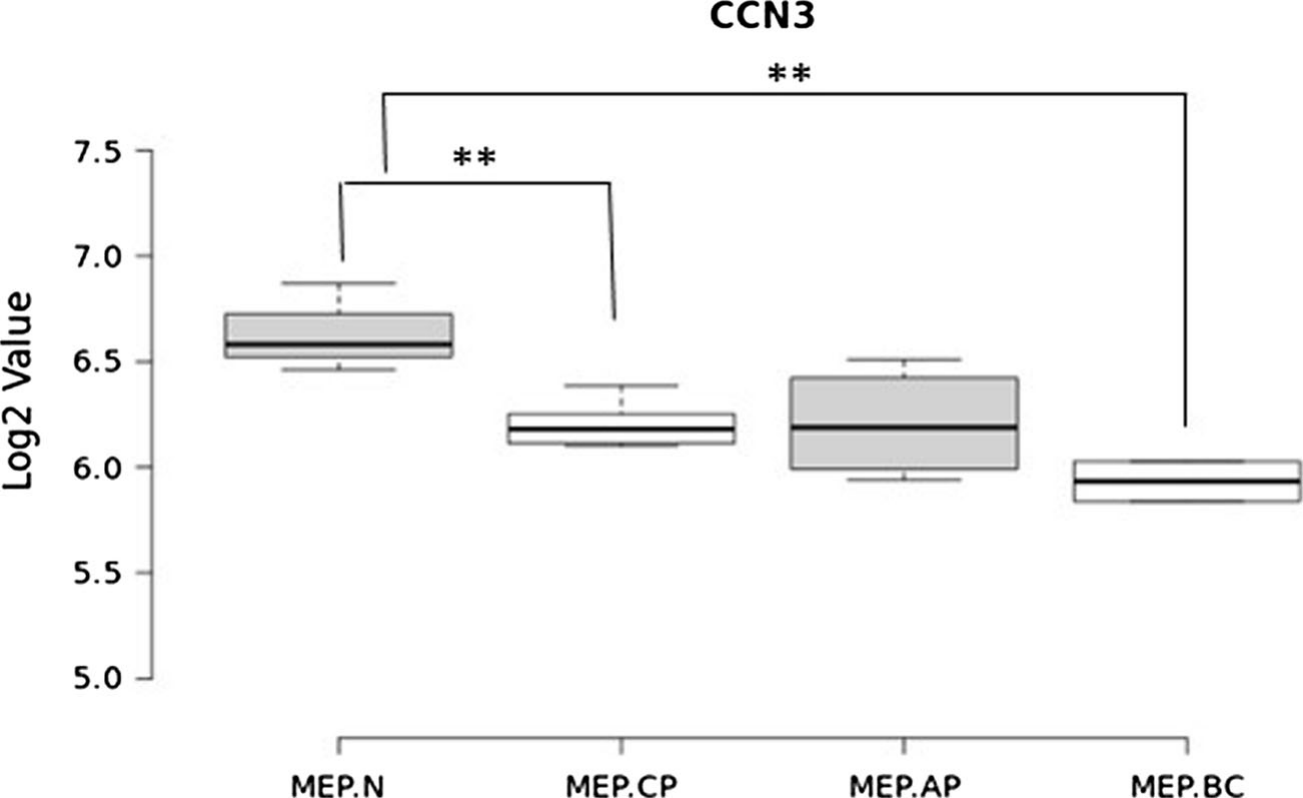
CCN3 expression in CML. CCN3 expression in megakaryocyte-erythroid (MEP) enriched cells from normal donors (N) and CML patients in chronic phase (CP), acute phase (AP) or blast crisis (BC). Data from GSE47927

CCN3 expression is dys-regulated at both the RNA and protein level in CML and there is a reciprocal relationship between CCN3 and BCR-ABL expression (McCallum et al. 2006). A comparison of CCN3 gene expression from purified stem and progenitor cells from CML patients in different stages of disease (12 samples) and normal volunteers (3 samples) demonstrated that there are significantly lower levels of CCN3 in megakaryocyte-erythroid progenitor (MEP) cells from CML patients compared to healthy counterparts (Cramer-Morales et al. 2013). Figure 4 we used a multi-potent haemopoietic cell line, FDCP-MIX, expressing BCR-ABL kinase to investigate the effects of the kinase in early haemopoietic stem cells. CCN3 was found to be downregulated and tyrosine phosphorylated as a consequence of BCR-ABL kinase activity in these cells. Human CML cell lines and primary cells from patients also expressed low or undetectable levels of CCN3. Treatment in vitro with the tyrosine kinase inhibitor imatinib decreased BCR-ABL kinase activity and increased CCN3. The latter observations were validated by clinical studies in which patients treated with imatinib showed a return to normal levels of CCN3 expression when they responded to treatment (McCallum et al. 2006).

The mechanism of action of CCN3-induced growth regulation has been investigated using a number of cell line models. In the FDCP-MIX CML cell line model it was shown that BCR-ABL kinase activity not only regulates transcriptional activity but also enhances CCN3 secretion by the cells (McCallum et al. 2006). Overexpression of CCN3 in a human CML cell line resulted in a reduction in clonogenicity, increase in apoptosis and reduction in phosphorylation of Erk2 (McCallum et al. 2012). This is consistent with previous reports of tumour cell survival being impacted by the Erk1/2 pathway (Balmanno and Cook 2009).

CCN3 is a non-canonical NOTCH ligand which is known to be a core signalling pathway in the haematopoietic system (Bigas et al. 2012). In CML cells modified to overexpress CCN3 or treated with exogenous recombinant CCN3 there was a significant decrease in NOTCH1 signalling (Suresh et al. 2013). Furthermore, silencing of BCR-ABL in a CML cell line reduced full length NOTCH and inhibited cleavage of the NOTCH intracellular domain leading to a decrease in downstream effector molecules cMyc and Hes1 (Suresh et al. 2013). This suggests that disruption of the NOTCH-CCN3 pathway may contribute to the pathogenesis of CML (Suresh and Irvine 2015).

### Association of other CCN family members with haematopoiesis and blood cancers

With the advent of high throughput technologies it is possible to screen for expression of thousands of genes in both cell lines and primary clinical material. CCN family members are now included on many microarray platforms and information on expression profiling is increasing exponentially. Two large online datasets discussed in this review, BloodSpot and MILE, are prime examples of information which suggests a wider involvement of CCN family members in blood cancer than currently appreciated but has not yet been validated (Kohlmann et al. 2008; Bagger et al. 2016). For example, in addition to the role of CCN1 in AML discussed above, the MILE study shows that CCN2 expression is significantly decreased compared to normal cells in two cytogenetic subtypes of AML (inv(16), t(15;17)) that are classified as belonging to a more favourable risk group. CCN3 displays a more variable expression pattern in AML. It is found to be increased across three different cytogenetic subtypes (normal karyotype, complex karyotype and t(8;21)) all belonging to different risk groups, and is observed to be decreased in AML patients with MLL translocations (Kohlmann et al. 2008). None of these observations have been followed up with protein and mechanistic investigations.

This review has focused on the role of CCN members in haematological malignancies where there is more than simply basic expression data available. The haematology research community is currently focusing on targeting the microenvironment in addition to the malignant stem cell to improve therapeutic efficacy. It is clear the CCN family will play a key part in these future developments.

## Abbreviations

ALL: acute lymphoblastic leukaemia
AML: acute myeloid leukaemia
BCR-ABL: breakpoint cluster region – Abelson tyrosine kinase fusion gene
BM: bone marrow
CB: cord blood
CD: clusters of differentiation
CFU: colony forming unit
CLP: common lymphoid progenitor
CML: chronic myeloid leukaemia
CMP: common myeloid progenitor
DMAP: Differentiation map
E: erythroid
G: granulocyte
GEMM: granulocyte- erythroid-macrophage- megakaryocyte
HSC: haematopoietic stem cell
IDDM: insulin dependent diabetes mellitus
IL-7: interleukin 7
LSK: Lin-, Sca+, c-kit + cells
LTC-IC: long term culture initiating cell
M: macrophage
MEG: megakaryocyte
MGUS: monoclonal gammopathy of undetermined significance
MILE: Microarray Innovations in Leukaemia
MM: multiple myeloma
MNC: mono nuclear cells
NBM: normal bone marrow
n PB: normal peripheral blood
SCF: stem cell factor
SMM: smouldering multiple myeloma
SYK: spleen tyrosine kinase
TPO: thrombopoietin
